# Only one independent genetic association with rheumatoid arthritis within the *KIAA1109-TENR-IL2-IL21 *locus in Caucasian sample sets: confirmation of association of *rs6822844 *with rheumatoid arthritis at a genome-wide level of significance

**DOI:** 10.1186/ar3053

**Published:** 2010-06-16

**Authors:** Jade E Hollis-Moffatt, Michael Chen-Xu, Ruth Topless, Nicola Dalbeth, Peter J Gow, Andrew A Harrison, John Highton, Peter BB Jones, Michael Nissen, Malcolm D Smith, Andre van Rij, Gregory T Jones, Lisa K Stamp, Tony R Merriman

**Affiliations:** 1 Department of Biochemistry, 710 Cumberland Street, University of Otago, Dunedin 9054, New Zealand; 2 Department of Medicine, 85 Park Road, University of Auckland, Auckland 1142, New Zealand; 3 Department of Rheumatology, Middlemore Hospital, Hospital Road, Auckland 1640, New Zealand; 4 Department of Medicine, 23A Mein Street, University of Otago, Wellington 6242, New Zealand; 5 Department of Medicine, Great King Street, University of Otago, Dunedin 9054, New Zealand; 6 Department of Medicine, Daws Road, Flinders Medical Centre and Repatriation General Hospital, Adelaide 5041, Australia; 7 Department of Surgery, Great King Street, University of Otago, Dunedin 9054, New Zealand; 8 Department of Medicine, Riccarton Avenue, University of Otago, Christchurch 8140, New Zealand

## Abstract

**Introduction:**

The single nucleotide polymorphism (SNP) *rs6822844 *within the *KIAA1109-TENR-IL2-IL21 *gene cluster has been associated with rheumatoid arthritis (RA). Other variants within this cluster, including *rs17388568 *that is not in linkage disequilibrium (LD) with *rs6822844*, and *rs907715 *that is in moderate LD with *rs6822844 *and *rs17388568*, have been associated with a number of autoimmune phenotypes, including type 1 diabetes (T1D). Here we aimed to: one, confirm at a genome-wide level of significance association of *rs6822844 *with RA and, two, evaluate whether or not there were effects independent of *rs6822844 *on RA at the KIAA1109-TENR-IL2-IL21 locus.

**Methods:**

A total of 842 Australasian RA patients and 1,115 controls of European Caucasian ancestry were genotyped for *rs6822844*, *rs17388568 *and *rs907715*. Meta-analysis of these data with published and publicly-available data was conducted using STATA.

**Results:**

No statistically significant evidence for association was observed in the Australasian sample set for *rs6822844 *(odds ratio (OR) = 0.95 (0.80 to 1.12), *P *= 0.54), or *rs17388568 *(OR = 1.03 (0.90 to 1.19), *P *= 0.65) or *rs907715 *(OR = 0.98 (0.86 to 1.12), *P *= 0.69). When combined in a meta-analysis using data from a total of 9,772 cases and 10,909 controls there was a genome-wide level of significance supporting association of *rs6822844 *with RA (OR = 0.86 (0.82 to 0.91), *P *= 8.8 × 10^-8^, *P *= 2.1 × 10^-8 ^including North American Rheumatoid Arthritis Consortium data). Meta-analysis of *rs17388568*, using a total of 6,585 cases and 7,528 controls, revealed no significant association with RA (OR = 1.03, (0.98 to 1.09); *P *= 0.22) and meta-analysis of *rs907715 *using a total of 2,689 cases and 4,045 controls revealed a trend towards association (OR = 0.93 (0.87 to 1.00), *P *= 0.07). However, this trend was not independent of the association at *rs6822844*.

**Conclusions:**

The *KIAA1109-TENR-IL2-IL21 *gene cluster, that encodes an interleukin (IL-21) that plays an important role in Th17 cell biology, is the 20^th ^locus for which there is a genome-wide (*P *≤ 5 ×10^-8^) level of support for association with RA. As for most other autoimmune diseases, with the notable exception of T1D, *rs6822844 *is the dominant association in the locus. The *KIAA1109-TENR-IL2-IL21 *locus also confers susceptibility to other autoimmune phenotypes with a heterogeneous pattern of association.

## Introduction

Genetic associations implicate aberrant activation and regulation of autoreactive T-cells as central to RA. In addition to the established human leukocyte antigen locus *DRB1*, other genes more recently confirmed (either through wide replication or combined analysis at a genome-wide level of significance, *P *≤ 10^-8^) as playing a role in the development of RA are the protein tyrosine phosphatase non-receptor 22 gene (*PTPN22*) [1], cytotoxic T-lymphocyte associated 4 (*CTLA4*) [2], an intergenic region on human chromosome 6 [3,4], signal transducer and activator of transcription 4 (*STAT4*) [5,6], the TNF receptor-associated factor 1 region (*TRAF/C5*) [3,7,8], CD40 [9,10], B-lymphocyte kinase (*BLK*) and the NF-kB family member c-Rel [11]. Aside from *HLA-DRB1 *and *PTPN22*, the effects are weak (odds ratio (OR) < 1.3). Most of these loci are also implicated as risk factors in other autoimmune phenotypes [12].

The *KIAA1109-TENR-IL2-IL21 *region has been associated with a number of autoimmune phenotypes including type 1 diabetes (T1D) [13], ulcerative colitis [14], Crohn's disease [15], celiac disease [16], Graves' disease (GD) [13], systemic lupus erythematosus (SLE) [17], psoriatic arthritis [18], and juvenile idiopathic arthritis [19] (Table 1). There have been several studies testing this region for association with RA in European Caucasian sample sets, with varying levels of supporting evidence (0.24 >*P *> 2.8 × 10^-4^) [6,12,20,21]. There is extensive linkage disequilibrium across the region, hampering fine-mapping efforts [13], however it is clear that there are two independent autoimmune associated regions within the *KIAA1109-TENR-IL2-IL21 *gene cluster. Here, we aimed to consolidate all available data on two SNPs independently associated with autoimmunity within the *KIAA1109-TENR-IL2-IL21 *gene cluster: *rs6822844 *(minor allele protective) and *rs17388568 *(minor allele susceptible), each into a single meta-analysis of association with RA that included previously published data, new genotype data from Australasia, and publicly-available data from the Wellcome Trust Case Control Consortium (WTCCC) [22].

**Table 1:** Summary table of SNPs described in the literature and linkage disequilibrium relationship with *rs6822844 *and *rs17388568*

	Location (dbSNP130)	r^2 ^in relation to SNP		Gene	Disease									
SNP		*rs6822844*	*rs17388568*		T1D	CeD	GD	UC	CD	JIA	PSA	PS	RA	SLE
*rs6822844*	123728871	1	0.07	-	[13,21] - -	[16,40,41] - -		[14,15,38] - -	[14,15] - -	[19] - -	[18] - -	[18] =	[6,12,21] -	
*rs13151961*	123334952	0.89	0.10	*KIAA1109 *(intron)		[16,20,40,41] - -		[14] - -	[14] -		[18] - -	[18] =		
*rs6840978*	123774157	0.73	0.13	-		[16,40,42] - -		[14,38] - -	[14] -					
*rs13119723*	123437763	0.65	0.07	*KIAA1109 *(intron)		[16,40,42] - -		[14,38] - -	[14] -					
*rs11938795*	123292459	0.53	0.16	-		[16,42] - -		[15] -	[15] - -					
*rs11734090*	123447563	0.53	0.16	*KIAA1109 *(intron)		[16,42] -								
*rs12642902*	123727951	0.45	0.22	-		[16,42] -								
*rs7684187*	123560609	0.44	0.19	*TENR *(intron)		[16,42] -					[18] - -	[18] -		
*rs907715*	123754503	0.41	0.22	*IL21 *(intron)										[17] - -
*rs17388568*	123548812	0.07	1	*TENR *(intron)	[13] +		[13] +						[12] =	
*rs2221903*	123758362	0.08	0.88	*IL21 *(intron)										[17] +
*rs3136534*	123589223	0.10	0.82	-	[13] +									
*rs4505848*	123351942	0.10	0.82	*KIAA1109 *(intron)	[43] +								[20] =	
*rs6836189*	123760791	-	-	*IL21 *(intron)	[13] +									

CD: Crohn's disease; CeD: Celiac's disease; GD: Grave's disease; JIA: juvenile idiopathic arthritis; RA: rheumatoid arthritis; T1D: type 1 diabetes; PS: psoriasis; PSA: psoriatic arthritis; SLE: systemic lupus erythematosus; UC: ulcerative colitis. The SNPs are arranged into separate LD blocks in relation to *rs6822844 *and *rs17388568. *Key to symbols: OR < 0.8, - -; OR 0.8 to < 1.0, -; no effect, =; OR > 1.0 to 1.2, +; OR > 1.2, +

## Materials and methods

### Study participants

The Australasian European Caucasian RA samples consist of 842 patients of whom 31% were male. For the RA patients for whom data were available, 81% (601/739) were rheumatoid factor (RF) positive, 68% (333/491) were anti-cyclic citrullinated peptide (CCP) antibody positive and 80% (657/820) carried the *HLA-DRB1 *shared epitope (SE). RA diagnosis was confirmed in all patients by a rheumatologist using the ACR criteria [23]. Patients were recruited from hospital outpatient clinics in the Auckland, Bay of Plenty, Wellington, Christchurch and Otago regions of New Zealand, and from Adelaide in South Australia. European Caucasian control subjects (n = 505) without RA were recruited from the Otago and Auckland regions of New Zealand and were all > 17 years of age. A further 610 controls recruited from the Otago region had been genome-wide scanned using the Affymetrix Genome-Wide Human SNP Array 6.0 [24]. These elderly controls were > 60 years of age and in good general health. All subjects provided informed written consent and ethical approval for this study was given by the New Zealand Multi-region Ethics Committee and the Lower South Ethics Committee, and the Research and Ethics Committee of the Repatriation General Hospital, Adelaide. Genomic DNA was extracted from peripheral blood samples using a guanidine isothiocyanate-chloroform based (RA patients and controls) or modified salting out (elderly controls) extraction method.

### Genotype generation

Study participants were genotyped for *rs6822844, rs17388568 *and *rs907715 *using TaqMan^®^, assays ID C_28983601_10, ID C_33129431_10 and ID C_8949748_10 (Applied Biosystems, Foster City, CA, USA) respectively. Imputed RA and control genotypes were obtained for *rs6822844*, *rs17388568 *and *rs907715 *from 100% of the WTCCC dataset (1,856 cases, 2,933 controls) using the publicly available WTCCC data [22] using the program IMPUTE [25] and HapMap (NCBI Build 36 (db126b)) CEU data as reference haplotype set. Of the Australasian case sample set, 99.1% of subjects for *rs6822844*, 99.1% of subjects for *rs17388568 *and 98.9% of subjects for *rs9077015 *were successfully genotyped and, for the 505 member control sample set, 97.4% of subjects for *rs6822844*, 99.4% of subjects for *rs17388568 *and 99.4% of subjects for *rs9077015 *were successfully genotyped. The remaining New Zealand control genotypes (n = 610) were obtained from the genome-wide data, with 100% successfully genotyped for *rs17388568 *and 99.6% imputed for *rs6822844 *and *rs907715*.

### Statistical analysis

Genotype data were managed using the BC|SNPmax system (Biocomputing Platforms Ltd, Espoo, Finland). Testing for departures from Hardy-Weinberg equilibrium, for the significance of any difference in minor allele frequencies between patients and controls, calculating odds ratios and conditional association testing was done using the PLINK software package [26]. Logistic regression analysis was applied to the Australasian case-control sample set to stratify data according to gender, RF, CCP and SE status using the STATA 8.0 data analysis and statistics software package (StataCorp, College Station, Texas, USA). Meta-analysis was done using the STATA 8.0 metan software package and cumulative *P- *values reported. The Mantel-Haenszel test was used to estimate the average conditional common odds ratio between these two independent cohorts and to test for heterogeneity between the groups. *P- *values from the North American Rheumatoid Arthritis Consortium (NARAC) study [3], which could not be combined using meta-analysis owing to unavailability of allele counts, were combined using Fisher's method [27].

## Results

**Figure 1:**
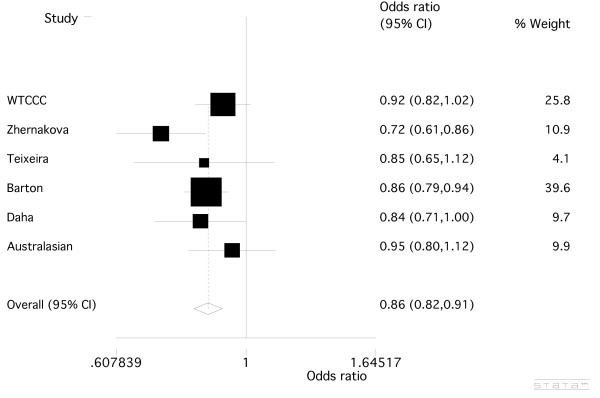
**Meta-analysis of the *KIAA1109-TENR-IL2-IL21 *SNP, *rs6822844***. Combined odds ratio values from data presented in Table 2. There was no evidence for heterogeneity between sample sets (*P *= 0.25).

We examined *rs6822844, rs17388568 *and *rs907715*. The latter SNP was chosen because it had been associated with risk of the systemic autoimmunity SLE in a single study [17] (OR = 0.78, *P *= 0.002), was in weak LD with both *rs6822844 *and *rs17388568 *and we hypothesized that it could represent a possible third effect within the *KIAA1109-TENR-IL2-IL21 *cluster, perhaps specific to systemic autoimmunity. The imputed genotype data from the WTCCC RA case-control sample revealed no significant association between *rs6822844 *and RA (Table 2; OR = 0.91 (0.82 to 1.02); *P *= 0.10) (the WTCCC samples did not overlap with those analysed in Barton *et al*. [12]). SNP *rs6822844 *had been genotyped in the NARAC sample set [3], revealing nominal evidence for a protective effect of the minor allele (Table 2; OR = 0.84 (0.74 to 0.96), *P *= 0.011). We then genotyped *rs6822844 *across the Australasian case-control sample set, finding no evidence for association between *rs6822844 *and RA (Table 2; OR = 0.95 (0.80 to 1.12), *P *= 0.54) although, consistent with the other association studies, the OR was less than one. Meta-analysis of all available data was undertaken (Figure 1). Zhernakova *et al*. [21] and Coenen *et al*. [28] both reported association of the *KIAA1109-TENR-IL2-IL21 *region with RA in overlapping Dutch case-control cohorts. We used data from the former study, as it was the only one to type *rs6822844*. The meta-analysis provided very strong (genome-wide) support for *rs6822844 *playing a role in the development of RA (OR = 0.86 (0.82 to 0.91), *P *= 8.8 × 10^-8^). The NARAC GWAS data (OR*_rs6822844 _*= 0.84 (0.74-0.96), *P *= 0.011) [7] were combined with the meta-analysis result, yielding *P *= 2.1 × 10^-8^.

**Table 2:** Association analysis of the *KIAA1109-TENR-IL2-IL21 *SNP *rs6822844 *with RA in seven European case control sample sets

	Genotype, no. (frequency)^1,2^			Minor allele, no. (frequency)	OR [95% CI]	*P*
Sample Set	1/1	1/2	2/2			
**WTCCC **[22]						
Case	1,242 (0.669)	553 (0.298)	61 (0.033)	675 (0.181)	0.91 (0.82 to 1.02)	0.10
Control	1,881 (0.642)	958 (0.326)	94 (0.032)	1,146 (0.195)		
**Zhernakova **[21]						
Case	748 (0.739)	243 (0.240)	21 (0.021)	285 (0.141)	0.72 (0.61 to 0.86)	2.0 × 10^-4^
Control	613 (0.663)	280 (0.303)	31 (0.034)	342 (0.185)		
**NARAC **[3]						
Case	NA	NA	NA	NA	0.84 (0.74 to 0.96)	0.011
Control	NA	NA	NA	NA		
**Teixeira **[20]						
Case	327 (0.753)	99 (0.228)	8 (0.018)	115 (0.132)	0.85 (0.65 to 1.12)	0.24
Control	313 (0.721)	110 (0.253)	11 (0.025)	132 (0.152)		
**Barton **[12]						
Case	2,739 (0.705)	1,052 (0.271)	95 (0.024)	1,242 (0.160)	0.86 (0.79 to 0.94)	5.4 × 10^-4^
Control	2,326 (0.674)	1,003 (0.290)	125 (0.036)	1,253 (0.181)		
**Daha **[6]						
Case	NA	NA	NA	285 (0.162)	0.84 (0.70 to 1.00)	0.051
Control	NA	NA	NA	325 (0.188)		
**Australasian**						
Case	583 (0.699)	221 (0.265)	30 (0.036)	281 (0.168)	0.95 (0.80 to 1.12)	0.54
Control	743 (0.674)	330 (0.299)	29 (0.026)	388 (0.176)		

(1) There were no significant departures from Hardy-Weinberg equilibrium, all *P *> 0.06

(2) Genotype data were imputed in the WTCCC sample set, and directly genotyped in all others

**Figure 2:**
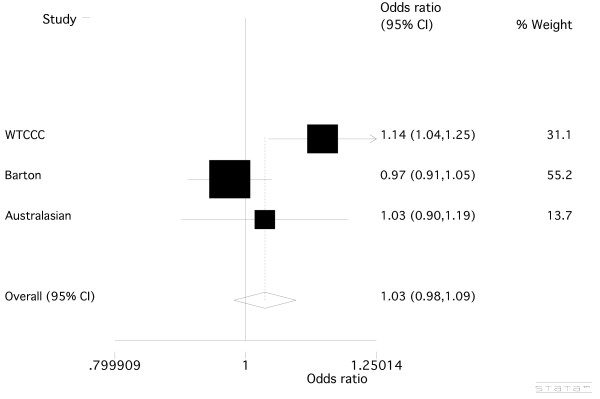
**Meta-analysis of the *KIAA1109-TENR-IL2-IL21 *SNP, *rs17388568***. Combined odds ratio values from the WTCCC [22], Barton *et al*. [12], and the Australasian case-control sample sets. There was evidence for heterogeneity between sample sets (*P *= 0.03)

**Figure 3:**
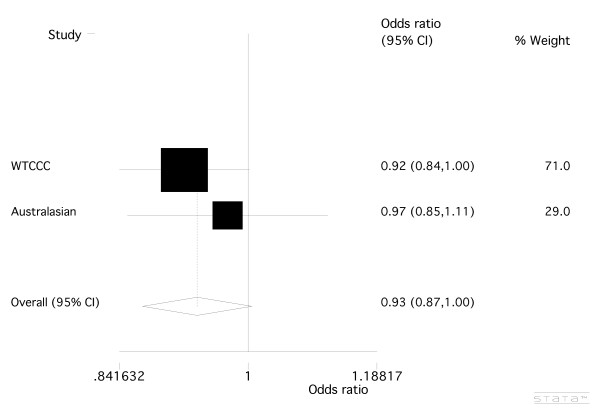
**Meta-analysis of the *KIAA1109-TENR-IL2-IL21 *SNP, *rs907715***. Combined odds ratio values from WTCCC [22] and the Australasian case-control sample sets. There was no evidence for heterogeneity between sample sets (*P *= 0.48).

For *rs17388568*, the genotype data from the WTCCC case-control sample revealed a weak association between *rs17388568 *and RA, with a susceptibility effect of the minor allele (Table 3; OR = 1.14 (1.04 to 1.25), *P *= 0.005). We genotyped *rs17388568 *across the Australasian case-control sample set, finding no evidence for association between *rs17388568 *and RA (Table 3; OR = 1.03 (0.90 to 1.19), *P *= 0.66), a result that was consistent with a study of a UK case-control sample set by Barton *et al*. [12] (Table 3; OR = 0.97 (0.91 to 1.05), *P *= 0.47). Meta-analysis of all the available data was done (Figure 2), with the combined analysis showing no significant role for *rs17388568 *in the development of RA (OR = 1.03, (0.98 to 1.09), *P *= 0.22). At *rs907715*, the imputed WTCCC genotype data also revealed a weak association (Table 3; OR = 0.92 (0.84 to 1.00), *P *= 0.055). Genotyping of the Australasian sample set and combined analysis with the WTCCC data (Table 3; Figure 3) slightly weakened the evidence for association of *rs907715 *with RA (OR = 0.93 (0.87 to 1.00), *P *= 0.07). Neither *rs17388568 *nor *rs907715 *nor any surrogate SNP was present in the NARAC data. Given the moderate LD between *rs907715 *and *rs6822844 *(Table 1), *rs907715 *was tested for association in the combined WTCCC and Australasian samples conditional on genotype at *rs6822844. *This revealed that the trend towards association seen for *rs907715 *was not independent of the association seen at *rs6822844 *(*P *= 0.38).

**Table 3:** Association analysis of the *KIAA1109-TENR-IL2-IL21 *SNPs *rs17388568 *and *rs907715 *with RA in three European Caucasian case-control sample sets

	Genotype, no. (frequency)^1,2^			Minor allele, no. (frequency)	OR [95% CI]	*P*
Sample Set	1/1	1/2^4^	2/2			
***rs17388568***						
**WTCCC **[22]						
Case	938 (0.505)	773 (0.416)	145 (0.078)	1,063 (0.286)	1.14 (1.04 to 1.25)	0.005
Control	1,610 (0.549)	1,119 (0.381)	204 (0.070)	1,527 (0.260)		
**Barton **[12]						
Case	2,057 (0.528)	1,530 (0.393)	308 (0.079)	2,146 (0.275)	0.97 (0.91 to 1.05)	0.47
Control	1,797 (0.516)	1,415 (0.406)	271 (0.077)	1,957 (0.281)		
**Australasian**						
Case	453 (0.543)	305 (0.366)	76 (0.091)	457 (0.274)	1.03 (0.90 to 1.19)	0.654
Control	584 (0.584)	461 (0.415)	67 (0.060)	595 (0.268)		
***rs907715***						
**WTCCC **[22]						
Case	835 (0.450)	822 (0.443)	199 (0.107)	1,220 (0.329)	0.92 (0.84 to 1.00)	0.055
Control	1,251 (0.427)	1,324 (0.451)	358 (0.122)	2,040 (0.349)		
**Australasian**						
Case	365 (0.438)	374 (0.449)	94 (0.113)	562 (0.337)	0.98 (0.86 to 1.12)	0.687
Control	468 (0.421)	524 (0.471)	120 (0.108)	764 (0.344)		

(1) There was one significant departure from Hardy-Weinberg equilibrium, *P *= 0.02 in Australasian cases

(2) Genotype data were imputed in the WTCCC sample set for *rs907715*, and directly genotyped in all others

The Australasian sample set was further examined by stratifying *rs6822844 *according to gender, RF, CCP and SE status (Table 4). This revealed no specific association to any particular sub-phenotypes analysed (*P *> 0.05). It should be noted that the power to detect association with sub-phenotype within the Australasian sample set was limited; for example, in the analysis with the largest amount of data available (RF) there was adequate power (> 70%) to detect an allele frequency difference between RF positive and negative cases only when the difference was equivalent to an OR > 1.5.

**Table 4:** Logistic regression analyses of the *rs6822844 *genotype frequencies in Australasian RA patients according to sub-phenotype

	*rs6822844*			
	GG	GT	TT	***P***
**Gender**				
Male	181 (0.69)	72 (0.28)	9 (0.03)	0.997
Female	352 (0.69)	139 (0.27)	17 (0.03)	
**Rheumatoid factor**				
Yes	419 (0.70)	155 (0.26)	22 (0.04)	0.834
No	95 (0.70)	38 (0.28)	3 (0.02)	
**anti-CCP**				
Yes	232 (0.70)	87 (0.26)	12 (0.04)	0.511
No	103 (0.66)	50 (0.32)	4 (0.03)	
**SE status**				
0	133 (0.69)	45 (0.28)	5 (0.03)	0.974
1 or 2	456 (0.70)	168 (0.26)	25 (0.04)	

Anti-CCP, anti-cyclic citrullinated peptide antibodies; NA, not available; RF, rheumatoid factor antibodies; SE, shared epitope status

## Discussion

Here we have combined published studies testing association of two SNPs, *rs6822844 *and *rs17388568*, located within the KIAA1109*-TENR-IL2-IL21 *region with RA: the former with two Dutch [6,21], one UK [12], and one Western Europe sample set [20]; and the latter with one UK sample set [12] - along with new Australasian data, and data from the WTCCC [22]. Meta-analysis (Figure 1) showed a consistent protective effect for the minor allele of *rs6822844 *with RA (OR = 0.86, *P *= 8.8 × 10^-8^). A similar protective effect was also observed in the NARAC GWAS for *rs6822844 *(OR = 0.84, *P *= 0.011), with combined evidence for association with RA of *P *= 2.1 × 10^-8 ^when combined with our meta-analysis *P- *value. Our analysis used the same European Caucasian RA sample sets recently meta-analysed by Maita *et al*. [29], with the addition of the WTCCC, Barton *et al. *[12], Australasian and NARAC [3] data. Meta-analysis of the T1D-associated SNP rs17385868 (Figure 2) on the other hand, did not reveal any significant association between the minor allele of *rs17388568 *and RA (OR = 1.03, *P *= 0.22). Note that SNP rs4505848, in strong LD with *rs17385868*, is more strongly associated with T1D (Table 1). Whilst there was weak evidence for association at *rs907715 *(Table 3), in a direction consistent with that previously observed in SLE [17], this effect appears to be dependent on *rs6822844*. When this paper was under review a genome-wide association scan meta-analysis in RA was published, in 5,539 autoantibody positive cases and 20,169 controls of European descent [30], with *P *= 7 × 10^-4 ^at *rs6822844*. We meta-analysed these data with data presented in Table 2 (removing the overlapping WTCCC samples), yielding OR = 0.86 (0.82 to 0.89), *P *< 1 × 10^-10^. The *rs6822844 *data provide compelling evidence supporting a role for the *KIAA1109-TENR-IL2-IL21 *locus in etiology of RA in Caucasian populations. Including the HLA region and the 10 loci confirmed by Stahl *et al*. [30] (note that Stahl *et al*. did not confirm *KIAA1109-TENR-IL2-IL21 *at a genome-wide level of significance), *KIAA1109-TENR-IL2-IL21 *is the 20^th ^locus associated with RA at a genome-wide level of significance (*P *≤ 5 × 10^-8^). The association at *rs6822844 *dominates at this locus, with no evidence for an independent effect at *rs17385868*, as is seen in T1D (Table 1) [13].

The *KIAA1109-TENR-IL2-IL21 *region was first implicated in autoimmunity after a GWAS in T1D [13,22] and has since been associated, also with a genome-wide level of support, with celiac disease [16] and ulcerative colitis [14] and, with lower supporting evidence, with SLE, psoriatic arthritis, Graves' disease and juvenile idiopathic arthritis [13,17-19] (Table 1). The region is characterized by a high degree of linkage disequilibrium [13], meaning that the underlying disease-causing variant(s) and gene(s) have not yet been determined. Collectively, these data point to at least two independent associations within the *KIAA1109-TENR-IL2-IL21 *region, with the pattern of association differing between autoimmune phenotypes (Table 1); one marked by *rs17388568 *(in the TENR gene) and the other by *rs6822844 *(which maps between IL-2 and IL-21). There is no appreciable linkage disequilibrium between *rs17388568 *and *rs6822844 *(r^2 ^= 0.07 in HapMap CEU samples). Different patterns of association are evident in the different autoimmune phenotypes. For example (referring to the risk conferred by the minor allele), susceptibility at *rs17388568 *and protection at *rs6822844 *is observed in T1D [13,21], some evidence for protection is seen at *rs17388568 *in Graves' disease [13], there is no evidence for association of *rs17388568 *(or markers in high LD) with RA (Figure 2) whereas *rs6822844 *confers protection (Table 1). The studies in Crohn's disease, ulcerative colitis, celiac disease, psoriatic arthritis, and JIA are consistent in reporting the *rs6822844*-mediated minor allele protective effect [14-16,18,19] (Table 1), with little data available on *rs17388568 *in comparison to *rs6822844*. The single study in SLE [17] did not include SNPs in LD with *rs6822844*, however there was evidence for a susceptibility effect at *rs17388568 *(using *rs2221903 *which is in strong LD with *rs17388568*, Table 1). Collectively, these studies point to heterogeneity at *rs17388568 *between RA and other autoimmune phenotypes (T1D, GD, SLE) with which RA shares other genes and clinical features.

Within the *KIAA1109-TENR-IL2-IL21 *gene cluster, IL-21 is of particular interest in the context of RA, and the Th1/Th17 axis in which IL-23R is involved. IL-21 is required for differentiation of naïve human CD4^+ ^T cells into Th17 cells [31], whereas IL-23 is critical in the expansion and maintenance of Th17 cells [32,33]. It is important to note that Th17 cells produce a variety of cytokines including IL-17A, IL-17F, IL-21 and IL-22. Human studies have demonstrated that IL-21 receptor (IL-21R)-positive cells are significantly increased in inflamed synovial tissues of RA patients compared to controls and that IL-21 enhances local T-cell activation, proliferation and proinflammatory cytokine secretion [34,35]. Alongside these findings, animal studies demonstrate that IL-21R deficient mice have normal T-cell and NK cell development but fail to develop spontaneous autoimmune disease suggesting that IL-21 plays a vital role in the development of autoimmune disease in rodents. Studies using arthritic mice and rats also demonstrate that inhibition of IL-21 expression correlates with modulation of serum IL-6 levels and improvements in disease severity [36]. However, it remains to be determined whether inhibition of IL-21 in humans with RA will have a similar beneficial effect given the significant differences between Th17 cell biology in mice and men.

Given the importance of Th17 cells in autoimmunity [37], the differential genetic effects observed in various autoimmune phenotypes mediated by the *IL23R *and *KIAA1109-TENR-IL2-IL21 *regions, evidence for genetic interaction between the *KIAA1109-TENR-IL2-IL21 *region and *IL23R *(in ulcerative colitis at least) [38], there are reasonable grounds for considering the hypothesis that genetic control of the Th1/Th17 axis is centered on cytokines (and their receptors) important in Th17 biology. It is important to note that not all RA patients have evidence of IL-17A within synovial tissue [39] and the role of IL-17A appears to be as an amplifier of inflammation rather than an absolute requirement for inflammation in RA. One possible explanation is genetic variation in the *KIAA1109-TENR-IL2-IL21 *locus that results in non-functional IL-21 and hence lack of IL-17A or vice versa. Regulation of this axis may be an important factor in determining the risk to particular autoimmune phenotypes, which may have implications for selection of targeted biological therapies within an individual. What will be important in understanding molecular control of autoimmunity will be association studies in large sample sets from different autoimmune phenotypes that comprehensively capture common variation in the *IL23R *and *KIAA1109-TENR-IL2-IL21 *loci, fine-mapping of the genetic effects and analysis of interaction between the disease-associated variants, both within and between loci.

## Conclusions

Genotyping of an Australasian RA case-control sample set, and meta-analysis with published and publicly-available data confirm at a genome-wide level of significance the rs6822844 SNP within the *KIAA1109-TENR-IL2-IL21 *locus to be a risk factor in RA (*P *= 2.1 × 10^-8^; OR = 0.86). There was no evidence for an independent effect on RA mediated by other variants within the *KIAA1109-TENR-IL2-IL21 *locus, as is seen in type 1diabetes.

## Abbreviations

*BLK*: B-lymphocyte kinase; CCP: cyclic citrullinated peptide; GD: Graves' disease; GWAS: genome-wide association scan; IL: interleukin; LD: linkage disequilibrium; NZ: New Zealand; OR: odds ratio; NARAC: North America Rheumatoid Arthritis Consortium; RA: rheumatoid arthritis; RF: rheumatoid factor; SE: shared epitope; SLE: systemic lupus erythematosus; SNP: single nucleotide polymorphism; *STAT4*: signal transducer and activator of transcription 4; T1D: type 1 diabetes; WTCCC: Wellcome Trust Case Control Consortium;

## Competing interests

The authors declare that they have no competing interests.

## Authors' contributions

JEH-M, MC-X and TRM planned the study design and oversaw its execution. RT provided technical and analytical support. JEH-M, MC-X, ND, PJG, AAH, JH, PBBJ, MN, MDS, AR, GJ and LKS took part in clinical recruitment and data acquisition. JEH-M, MC-X, RT, ND, PJG, AAH, JH, PBBJ, MN, MDS, AR, GJ, LKS and TRM prepared the manuscript.
